# Stability of GO Modified by Different Dispersants in Cement Paste and Its Related Mechanism

**DOI:** 10.3390/ma11050834

**Published:** 2018-05-18

**Authors:** Wu-Jian Long, Changle Fang, Jingjie Wei, Haodao Li

**Affiliations:** Guangdong Provincial Key Laboratory of Durability for Marine Civil Engineering, Shenzhen Durability Center for Civil Engineering, College of Civil Engineering, Shenzhen University, Shenzhen 518060, China; 2150150420@email.szu.edu.cn (C.F.); 2150150416@email.szu.edu.cn (J.W.); lihaodao2017@email.szu.edu.cn (H.L.)

**Keywords:** graphene oxide, dispersing agents, agglomeration, cement paste, mechanism

## Abstract

Graphene oxide (GO) is a potential material to be used as a nano-reinforcement in cement matrix. However, a prerequisite for GO to fulfill its function in the cement matrix is homogeneous dispersion. In this study, the effects of three different dispersing agents (DAs), including polycarboxylate-based high range water reducer (P-HRWR), naphthalene-based high range water reducer (N-HRWR), and air entraining agent (AEA) on the dispersion of GO in aqueous solution, simulated concrete pore solution (SCPS), and suspension of cement pastes were sequentially investigated. Results showed that the dispersion effect of GO in aqueous solutions was improved with different DAs. However, the homogeneous dispersion of GO in aqueous solution re-agglomerated in SCPS and suspension of cement pastes. It was concluded that as the cement content and pH of aqueous solutions increased, GOs re-agglomerated and precipitated in an alkaline solution. A possible mechanism was proposed in this study and it was believed that electrostatic interactions and steric hindrance provided by the P-HRWR further made GOs stable in aqueous solutions. The ions and pH of cement pastes increased with the increasing amount of cement, which caused the separation of P-HRWR from GOs. Therefore, GOs were re-agglomerated and absorbed on the surface of the cement particles, resulting in GOs sedimentation.

## 1. Introduction

Modern concrete technology is shifting towards a high-performance application with a desire to be sustainable [1,2]. Nano-materials have shown to improve the micro-structure and comprehensive performance of cement-based composites [3,4,5,6]. Moreover, several studies on self-sensing cement composites which uses nanocomposites have also been reported [7,8,9]. Thus, nano-modified cement-based materials are the main technical ways for developing ultra-high-performance concrete (UHPC). However, due to small particle size, large specific surface area and high surface energy of nano-materials, the use of nano-materials in cement matrix causes high water consumption and agglomeration [10,11,12,13]. In recent years, the development of nano-carbon-based materials (carbon nano-tubes, graphene, graphene oxide, etc.) and zero-dimensional nanoparticles (nano-SiO_2_, nano-CaCO_3_, nano-Al_2_O_3_, etc.) has demonstrated that the microstructure and mechanical performance of concrete can be improved significantly [14,15,16]. 

Graphene oxide (GO) is characterized by a high aspect ratio and good interface contact with cement matrix [17,18]. GO can further facilitate the generation of hydration products and reduce the porosity of hardened cement pastes allowing for improved strength, toughness, and durability of the cement-based materials [19,20]. However, a high amount of GO in cement matrix does not necessarily give a better performance because an excessive amount of GO results in a loss of mechanical performance [21]. In addition, a high alkalinity and high ionic strength of concrete can result in agglomeration of GO [22].

The uniform dispersion and distribution of GO in cement pastes is essential to fulfill the function of GO. When GO is non-uniformly dispersed, their sizes may not be closer to those of calcium silicate hydrate (C–S–H) gel, and thus cement matrix at the nano-scale could not be improved [2]. In current studies, the uniform dispersion of GO is achieved by physical and chemical methods [23,24,25,26]. The common physical dispersion methods include ultrasonication, ball milling, and high shear mill [23]. The chemical dispersion methods usually use surfactants for surface activation, such as polycarboxylate-based high range water reducer (P-HRWR) and naphthalene-based high range water reducer (N-HRWR) [24,25,26].

Currently, most studies focus on the dispersion of GO in aqueous solutions [19,27,28], and rarely mention its dispersion or re-agglomeration after being added into cement pastes. However, a homogeneous dispersion of GOs in aqueous solutions may not be a guarantee of a uniform dispersion in cement pastes [29,30]. Due to the complex electrolyte, the dispersion and stability of GO in the pore solution of concrete can be very different from those in aqueous solutions. After studying the dispersion conditions of carbon nano-fibers under different dispersing agents and different pH, Stephen et al. [24] pointed out that, no matter when the carbon nano-fibers were dispersed in an aqueous solution or a simulated concrete pore solution (SCPS), P-HRWR always had the optimum dispersion effect for carbon nano-fiber in comparison to N-HRWR and air entraining agent (AEA). P-HRWR further weakened the re-agglomeration effect of carbon nanotube fiber in hardened pastes. The study done by Kaili et al. [31] showed that N-HRWR had a better dispersion effect for graphene. When the mass of N-HRWR and graphene were the same, the most stable dispersion was witnessed in aqueous solutions. Therefore, different types of water reducer and concentrations exert different influences on the dispersion of nanocarbon-based materials in aqueous solutions. In addition, the change of pH in the process of cement hydration also influences the dispersion of GO. It is known that the pH of the pore solution of concrete is 12.6 to 13.5 [32,33]. Meanwhile, GO in pore solutions with high pH and high ionic strength prefers to yield agglomeration and sedimentation [32,34]. 

Based on the abovementioned, this paper adopts three different dispersing agents (P-HRWR, N-HRWR, and AEA) and three different GO to DA mass ratios (1:1, 3:1, and 9:1) to investigate the dispersion, re-agglomeration and related mechanisms of GO in aqueous solutions, SCPS and suspension of cement pastes. Understanding the behavior of GOs in aqueous solutions and the relationship between the relative re-agglomeration state of GOs in SCPS and in cement pastes is critical for the development of reliable GO cement composites.

## 2. Materials and Methods

### 2.1. Raw Materials

#### 2.1.1. Graphene Oxide (GO)

The suspension of GO (3 g/L) was prepared by dispersing the graphite oxide powder into water with probe sonication. Graphite oxide was purchased from the Sixth Element Ltd. (Changzhou, China) and the properties were given in Table 1. Based on the previous study [35], the power and time of ultrasonic were selected 25 Hz, 400 W and 2 h (2 s on, 4 s off), respectively. 

**Figure 1:**
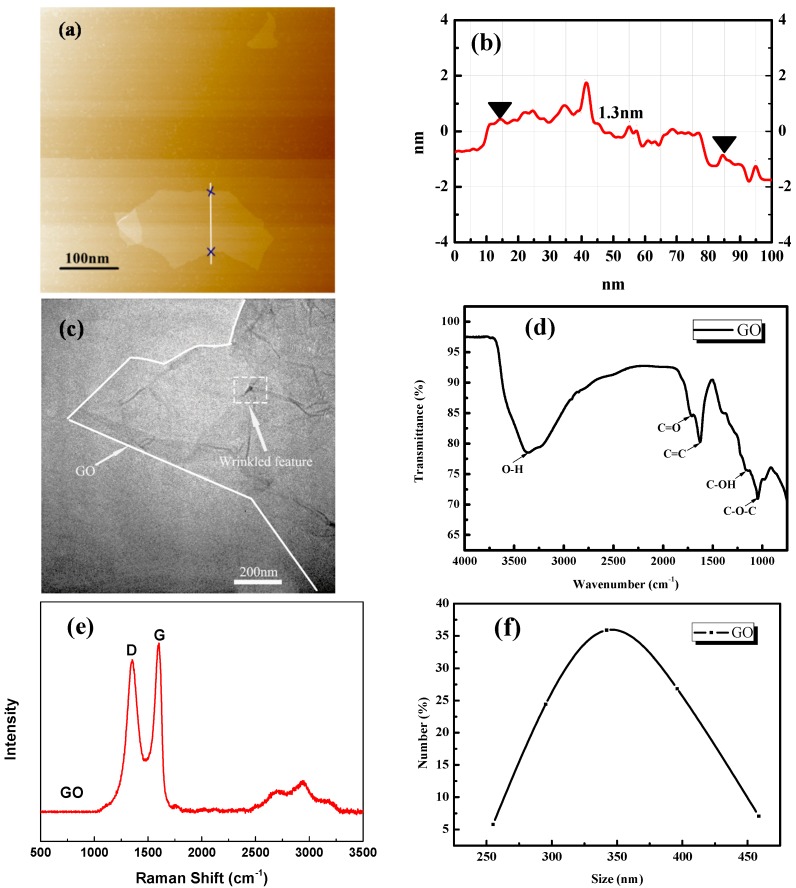
The characterization of GO (**a**) AFM image; (**b**) AFM spectra; (**c**) TEM Image; (**d**) FTIR transmittance spectra; (**e**) Raman spectra; (**f**) LPSA image.

The GO sample was characterized using atomic force microscope (AFM, type ICON-PT-PKG, Bruker, Fremont, CA, USA), transmission electron microscopy (TEM, type JEM-1230, NIPPON TEKNO company, Osaka, Japan), as illustrated in Figure 1a–c. AFM image (Figure 1a) showed that the used GO sheets exhibited irregular shapes with a dimension of about 0.1 μm and a thickness of about 1.3 nm. TEM (Figure 1c) image demonstrated that GO was an almost transparent nanosheet with various wrinkles and folds.

The oxygen-carbon groups of GO were tested with Fourier transform infrared spectroscopy (FTIR, Perkin Elmer Spectrum 100, Perkin Elmer Company, Waltham, MA, US), as shown in Figure 1d. The bands that appeared at about 3400 cm^−1^ for GO were assigned to the –OH stretching vibration because of the existence of hydrolytic groups and residual water [36]. An absorption peak for the C=C stretch was observed at 1634 cm^−1^ and peaks at 1720 cm^−1^ were also seen in carbonyl groups (C=O), in agreement with the peak at 1058 in the –COOH group indicating alkoxy C–O stretching [37,38]. These oxygen functionalities endowed GO with a high hydrophilicity, thus making it easily dispersed in the aqueous cement pastes. GO took up a large specific surface area, which provided a significantly large contact area with the cement materials. The chemical composition of GO was consisted of 49.6 wt % C, 2.1 wt % H and 48.3 wt % O (by difference), respectively.

The Raman spectrum (RM 3000 Micro-Raman system (Bruker, Fremont, CA, USA)) of GO was also conducted, as shown in Figure 1e. There were two main Raman shifts characterized by carbon nano-materials ranging from 1200 to 1700 cm^−1^. The first band at 1620 cm^−1^ could be attributed to the graphite mode (G band), while the second band at 1380 cm^−1^ could be attributed to diamondoid mode (D band) [39]. In comparison with graphite, the ID/IG mass ratio was observed to rise, with the presence of disordered structure in graphite arising from different functional groups in the structure [40,41].

The particle size distribution of well dispersed GO was characterized by laser particle size analyzer (LPSA, Mastersizer 2000, Malvern Panalytical, City of Malvern, UK), as shown in Figure 1f. The particle size distribution ranged from 255 nm to 459 nm and mostly distributed in 350 nm.

#### 2.1.2. Dispersing Agents (DAs)

A sulfonated-naphthalene condensate high-range water reducer (N-HRWR) type of Sika KS-20, a polycarboxylate-based HRWR (P-HRWR) type of Sika TMS-YJ-1, and an air-entraining admixture (AEA) of BASF MicroAir (BASF SE, Ludwigshafen, Germany) were employed, which conformed to the requirements of GB 50119-2013 [42]. P-HRWR was blended by the type of RMC-3 and CP-WRM50 with a ratio of 1/4. The characteristics of DAs are listed in Table 2. These three different DAs were thus selected for comparisons based on their different mechanisms of action and known compatibility with cement hydration. The N-HRWR is an anionic surfactant that works through electrostatic repulsion by providing particles with a highly negative surface charge through its adsorption onto their surface, causing their mutual repulsion [43]. The P-HRWR is a polymer surfactant that works through a dual mechanism of electrostatic repulsion and steric stabilization in which the long molecules of the organic polymer wrap around the particles to prevent their aggregation by physically precluding them to approach each other [43,44]. The AEA is a modified resin acid compound-based anionic surfactant that lowers the surface tension of water [45]. HRWRs are frequently used in concrete technology to improve the workability of cementing materials system, and AEAs are widespread practice to increase freeze-thaw and scaling resistances.

#### 2.1.3. Cement

**Figure 2:**
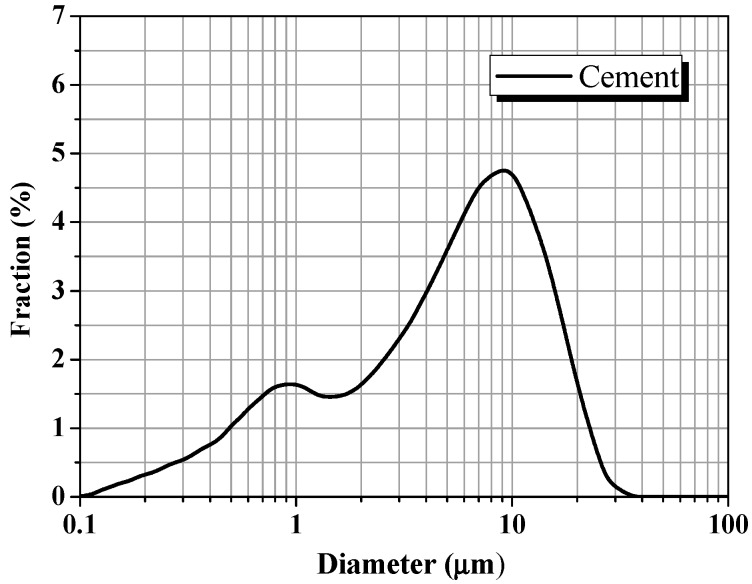
Distribution of particle diameter of cement.

Type I ordinary Portland cement, conforming to the requirements of Chinese Standard GB 175 [46], was used as the base material in this research. Figure 2 shows the particle size distribution of cement and the chemical composition and physical properties of cement is detailed in Table 3. 

### 2.2. Preparation of GO Suspensions

GO suspensions were prepared to determine the adequate dispersion conditions with regards to DA type as well as DA to GO mass ratio. GO suspensions were also employed to monitor the stability of the dispersions as a function of pH in dynamic conditions close to those obtained during cement mixing.

#### 2.2.1. DA Type and DA to GO Mass Ratio 

In order to achieve the homogeneous and stable dispersions of GO, the effects of DA type (N-HRWR, P-HRWR, and AEA) and DA to GO mass ratios (1:1, 3:1, and 9:1) on GO dispersion in three solutions were investigated, respectively. These three solutions were aqueous solution, SCPS, and suspension of cement pastes. The DA to GO mass ratios of 1:1, 3:1, and 9:1 were chosen based on preliminary results, consistent with the limited data found in the literature [47,48,49,50], which suggested DA to carbon nano-material mass ratios ranged from 4:1 to 8:1 for effective dispersion.

In order to analyze the UV–Vis absorbance of the GO solutions, the GO aqueous solution was used at a concentration of 0.03 g/L, which was obtained by 100 times the dilution of well-dispersed 3 g/L GO aqueous solution. 

Due to the functions of different dispersing agents, GO exhibited diverse stability by adding different dispersing agents. This stability can then be used to present speed of re-agglomeration and sedimentation of GO over time. When GO in the solution began to precipitate, the concentration of GO solutions decreased, and then the absorbance of GO decreased as well. By analyzing how the absorbance of the upper solutions differed over time, the effect of different DAs on the dispersing effects of GO in aqueous solutions could be observed. Similarly, the dispersing effects of GO in aqueous solution can then be examined with various DAs to GO mass ratio. The specific surface area of GO suspension after 30 min of resting was also tested in order to further analyze the influences of different dispersant types and concentration on the dispersing effects of GO in aqueous solutions. Table 4 illustrates DA to GO mass ratios and the corresponding DA to solution mass ratios for different GO concentration. 

Optimum DA to GO mass ratios were chosen depending on the best dispersion of GO aqueous solution. Only this optimum DA to GO mass ratio was then considered when GO were dispersed in SCPS and cement pastes. The SCPS presents the typical chemical environment of hydrating Portland cement pastes at an age of 2 h with a composition as reported in Ref. [51]. Occurrence of GO agglomeration at this stage will most likely remain at later stages of hydration during the hardening process. The SCPS was prepared with deionized water and 20.2 g/L potassium hydroxide (KOH), 1.2 g/L sodium hydroxide (NaOH), and 21.4 g/L calcium sulfate hemihydrate (CaSO_4_·½H_2_O), resulting in a measured pH about 13.3.

#### 2.2.2. Effect of Cement Addition and Solution pH 

Aqueous solution suspensions of GOs (0.03 g/L) at different DA to GO mass ratios (1:1, 3:1, and 9:1) were prepared. To account for the dynamic conditions obtained during cement mixing, cement powder was added to the suspensions in 0.34 g increments per 100mL of suspension to cover a solution pH ranging from about 7 (pH of the GO aqueous solution with DA) to 11.5 to 12 (pH of cement pastes). The stability of the dispersion as a function of pH was visually monitored.

### 2.3. Testing Methods

#### 2.3.1. UV–Vis Absorption Spectrum 

**Figure 3:**
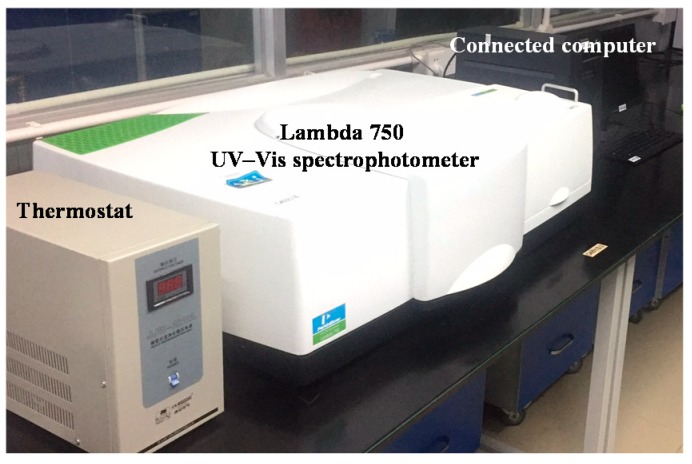
Lambda 750 UV–Vis spectrophotometer.

Ultraviolet–visible spectrum can be generated when ultraviolet light, visible light and near-infrared light are absorbed by materials. The spectrum can be used to analyze the absorbance of the materials. The absorption degree is proportional to the concentration of the materials. The larger the absorbance of the solution, the more homogenous dispersion of GO in the solution [52,53,54]. As an indirect method, UV–Vis measures the degree of dispersion of GO in a non-quantitative manner. The dispersion of the materials are quantitatively reflected by the spectrum and better dispersion measured means higher absorbance. In this work, Lambda 750 UV–Vis spectrophotometer (PerkinElmer, Waltham, MA, USA, see in Figure 3) is used to evaluate the adsorption performance of GO aqueous solution.

#### 2.3.2. Characterization of the Specific Surface Area of GO

Low Field Nuclear Magnetic Resonance (LF-NMR, PQ001 type, Niumag Company, Shanghai, China) can measure and analyze the specific surface area of particles in a suspension state. When the particles are in the state of suspension, a layer of water molecules with a thickness of L is adsorbed on the surface of particles. It was called the adsorption water and outside the molecular layer of which was the free water. The activity of H proton in the adsorption water and that of free water are very different, making a relaxation time of the adsorbed water far less than free water. This difference can reflect the amount of adsorbed solution on the surface of the particles and thus the wet specific surface area of the particles is derived.

**Figure 4:**
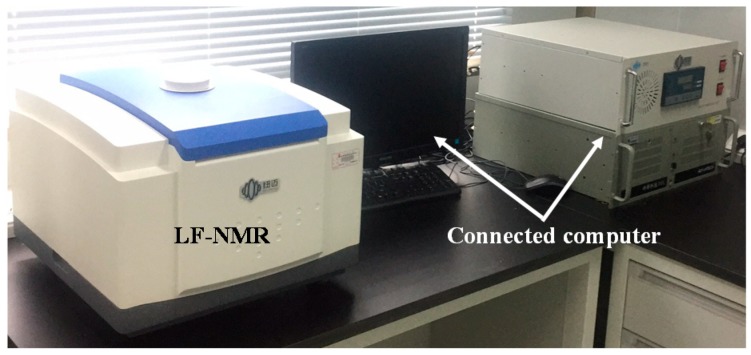
Particle size surface area analyzer.

LF-NMR characterization of GOs dispersions was performed by means of a particle size surface area analyzer given in Figure 4. The samples were vacuumed at 3 mTorr and heated to 80 °C for 5 min prior to analysis. The testing process was divided into two steps: first a solvent (distilled water) measurement was conducted, followed by a sample measurement. 

## 3. Results and Discussion

### 3.1. Dispersion State of GO in Aqueous Solutions

#### 3.1.1. Selection of Wavenumber

**Figure 5:**
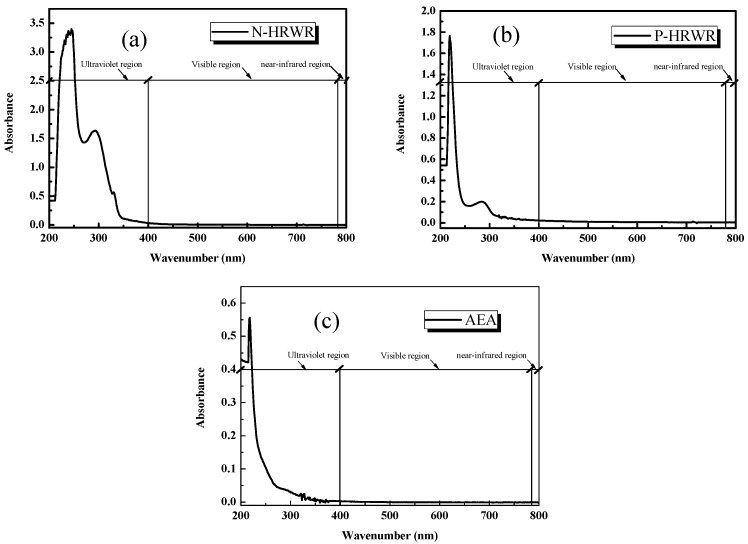
Typical UV–Vis absorption spectra of different DAs solution: (**a**) N-HRWR; (**b**) P-HRWR; (**c**) AEA.

Figure 5 presents the absorbance curves of different DAs at the same concentration (0.03 g/L) with wavenumbers of 200 to 800 nm (the wavenumber of ultraviolet light spectrum is under 400 nm, the wavenumber of visible light spectrum is from 400 to 780 nm and the wavenumber from 780 to 800 nm is near-infrared spectrum). Unstable absorption could be observed in the ultraviolet area with the characteristic peaks of each DA. Two peaks of N-HRWA appeared at 245 nm and 293 nm; two peaks of P-HRWR occurred at 219 nm and 284 nm; one peak of AEA was at 281 nm. These three DA solutions remained stable and exhibited absorption within the range of 400 to 800 nm. Based on the above analysis, while conducting the absorbance test of aqueous suspensions of GO, the excitation wavenumber was set to 650 nm, which should help eliminate the absorption influence of DAs and ensure the change of absorbance was due to dispersion of GO [53].

#### 3.1.2. Effect of DA Types

**Figure 6:**
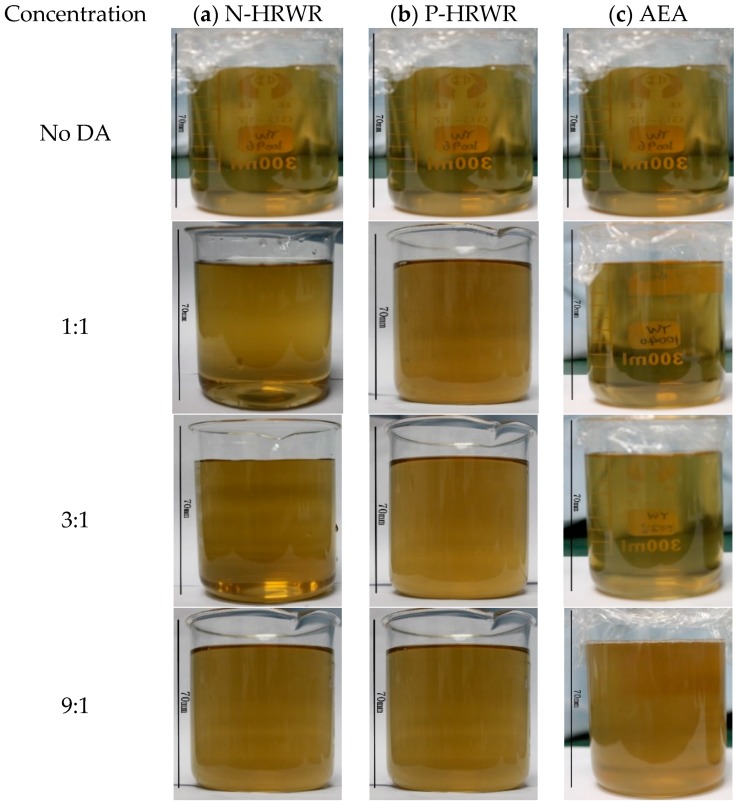
Dispersion state of GO suspensions (0.03 g/L) in water with different DAs and concentration: (**a**) N-HRWR; (**b**) P-HRWR; (**c**) AEA.

**Figure 7:**
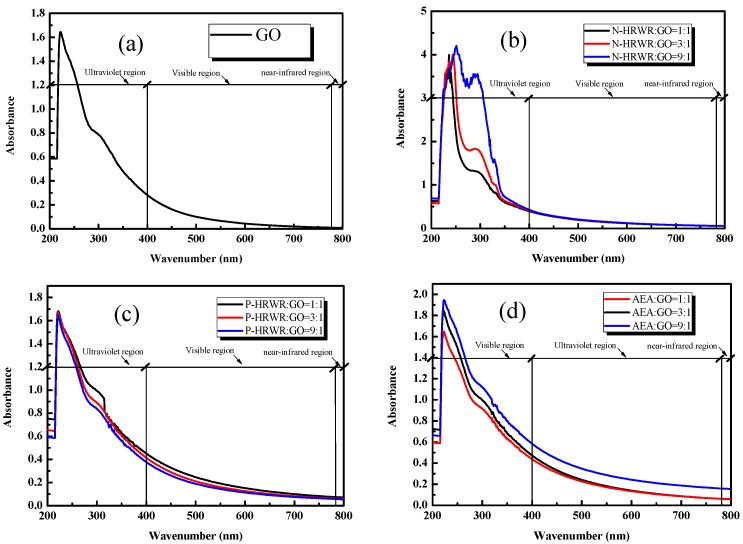
Absorption spectra of GO solution with different DAs and concentration: (**a**) without DAs; (**b**) with N-HRWR; (**c**) with P-HRWR; (**d**) with AEA.

**Figure 8:**
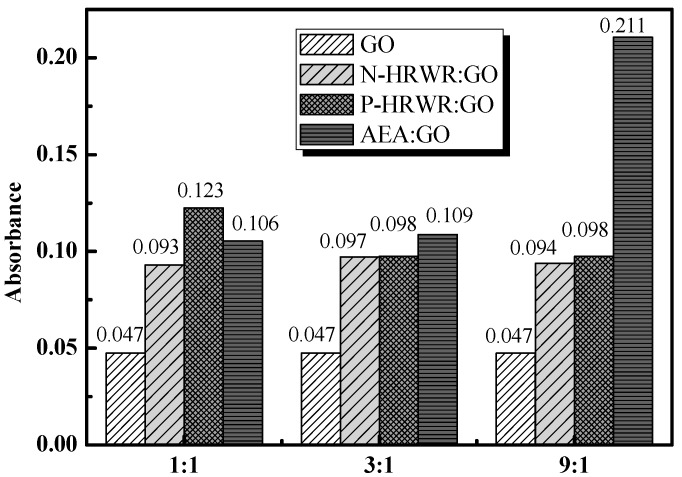
Different absorbance on 650nm of GO solution with different DAs and concentration.

Figure 6 shows the GO solution with the different DAs and different DA to GO mass ratios after sonication. It can be seen that, after adding N-HRWR and P-HRWR into the aqueous solution of GO, the originally clear aqueous solution immediately became turbid. In the case of AEA to GO mass ratio, the clear aqueous solution did not turn turbid until the ratio reached its maximum value of 9:1. To further determine the influence of different dispersing agent types on the dispersion of GO in aqueous solutions, an UV–Vis spectrophotometer was used to test solutions of different dispersion types, as shown in Figure 7. As shown in Figure 8, the three dispersing agents were all able to facilitate the dispersion of GO in aqueous solutions. When N-HRWR: GO = 3:1, the absorbance of the aqueous solution of GO at 650 nm exhibited the maximum value of 0.098; when P-HRWR: GO = 1:1, the absorbance of the aqueous solution of GO at 650 nm exhibited a maximum value of 0.123; when AEA: GO = 9:1, the absorbance of the aqueous solution of GO at 650 nm reached its maximum value of 0.211. Thus, the higher the absorbance of the solution, the more uniform the dispersion of GO in the solution [52,53]. Thus, when AEA: GO = 9:1, AEA, among the three dispersing agents, realized the optimum dispersion for GO in the aqueous solution.

#### 3.1.3. Effect of DA Concentrations

As shown in Figure 7, after adding N-HRWR and P-HRWR into the aqueous solution of GO, the originally clear aqueous solution immediately became turbid and the increase of the concentrations of N-HRWR and P-HRWR in the aqueous solution of GO remained turbid. However, the aqueous solution of GO was always stable, and did not present any agglomeration or sedimentation of GO. When AEA: GO was 1:1 or 3:1, the aqueous solution of GO still remained in a clear state; however, when AEA: GO was increased to 9:1, the originally clear aqueous solution became turbid. According to the results of Figure 8, with the increase of N-HRWR to GO mass ratio from 1:1, 3:1, to 9:1, the absorbance of the aqueous solution of GO remained unchanged. That is, increasing N-HRWR to GO mass ratio provided no benefit for the dispersion of GO in the aqueous solution. With the increase of P-HRWR to GO mass ratio from 1:1 to 3:1, the absorbance of the aqueous solution of GO gradually declined, and increasing from 3:1 to 9:1 the absorbance of the aqueous solution of GO remained unchanged. Thus, within a certain range, increasing P-HRWR to the GO mass ratio would be detrimental to the dispersion of GO in the aqueous solution. The optimum P-HRWR to GO mass ratio would be 1:1. With the increase of AEA to GO mass ratio from 1:1, 3:1 to 9:1, the absorbance of the aqueous solution of GO gradually increased. Thus, increasing AEA to GO mass ratio would be conducive to the dispersion of GO in the aqueous solution and the optimum AEA to GO mass ratio would be 9:1.

As shown in Figure 8 and Table 5, the greater the absorbance of GO, the larger the specific surface area of the GO in the solution. It shows that the dispersing agents can effectively prevent the re-agglomeration of GO in the aqueous solution. It was difficult to distinguish the re-agglomeration of GO by visual inspection when the GO aqueous solution was rested for 30 min.

### 3.2. Dispersion State of GO in Sumulated Concrete Pore Solution (SCPS)

**Figure 9:**
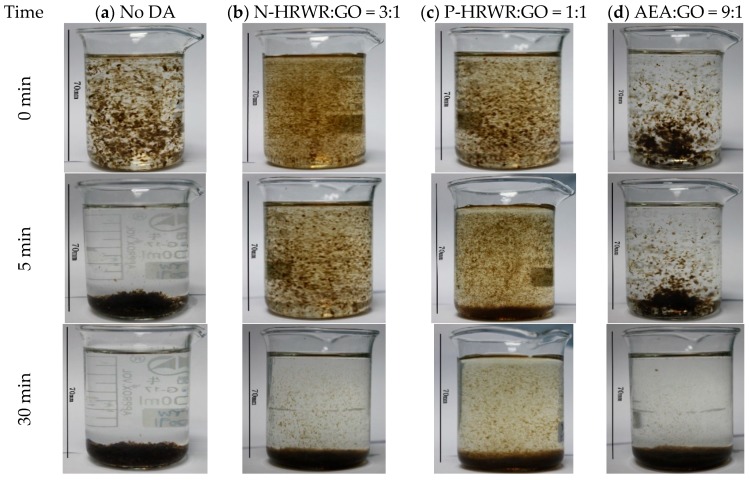
Dispersion state and stability of GO suspensions in SCPS with best DA to GO mass ratio: (**a**) no DA; (**b**) N-HRWR; (**c**) P- HRWR; (**d**) AEA.

Figure 9 simulates the dispersion state of GO in SCPS, in which the GO to DAs mass ratio is based on the best GO dispersion in aqueous solutions. It was observed that GO exhibited appreciably greater agglomeration no matter whether DAs were present or not. When there was no DA, almost all the GO separated from SCPS mixed solution after 30 min, resulting in sedimentation. A few remaining GO suspensions were seen when DAs were present. 

The specific surface area of the upper solution of GO in the SCPS after 30 min of resting is shown in Table 6. The re-agglomeration and sedimentation of GO were observed without dispersing agents. The AEA did not prevent the re-agglomeration of GO in the SCPS, while N-HRWR and P-HRWR could partially prevent GO from re-agglomeration. Further, the effect of P-HRWR was more obvious than that of N-HRWR.

These results provided the evidence that dispersing agents had a positive effect on GO dispersion in SCPS. During the dispersion process of GO in SCPS solution, cations in SCPS (i.e., Ca^2+^, Na^+^, K^+^, etc.) competed with GO for polymers and combined with GO at the same time, which resulted in sedimentation [22,55].

### 3.3. Dispersion State of GO in Cement Paste

#### 3.3.1. Effect of Cement Addition on GO Dispersion

**Figure 10:**
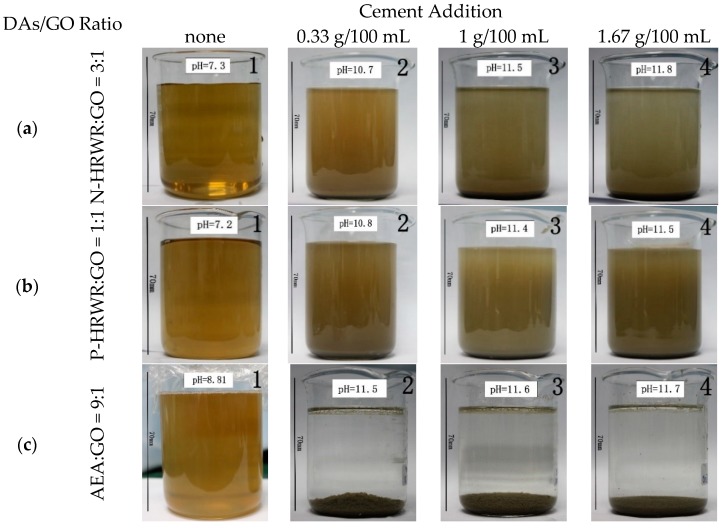
Effect of cement addition on the stability of GO dispersion in aqueous solution at 5 min.

A decrease in dispersion quality of the GO suspensions was observed with increasing cement addition. The separation of the GO agglomerates from solution and then the formation of GO agglomerations was seen when the suspension pH reached a value of 11 or higher. As shown in Figure 10(a_3_,a_4_,b_3_,b_4_) the top of the solution became light in color compared to Figure 10(a_1_,a_2_,b_1_,b_2_) respectively. In Figure 10(c_2_,c_3_,c_4_), the solution turned colorless, and GO uniformly experienced agglomeration and sedimentation. These results agreed with the findings reported by Mendoza and Grunlan et al. [56,57], where a decrease in dispersion as a function of increasing pH has been reported for CNTs. According to Figure 10a,b, N-HRWR and P-HRWR were conducive to the dispersion of GO in cement pastes, even when the pH was greater than 11 compared to AEA. The cement pastes with N-HRWR and P-HRWR still had a majority of GO which did not experience sedimentation, and the solution with N-HRWR (Figure 10(a_4_)) was lighter in color at the top than that with P-HRWR (Figure 10(b_4_)). Moreover, when the pH exceeded 11, the GO in the cement pastes added with AEA had experienced full agglomeration and sedimentation (Figure 10(c_2_,c_3_,c_4_)). The abovementioned results illustrated that P-HRWR was the most conducive to GO dispersion in cement pastes among the three DAs. Furthermore, these results inferred that homogeneous dispersion in the aqueous solution was not a guarantee of effective dispersion in the cement pastes.

The specific surface area of the upper solution of GO mixed with various dispersants in the cement suspension (cement content of 1.67 g/100 mL) was given, as shown in Table 7. It was concluded that the three dispersants of P-HRWR, N-HRWR and AEA had the promoting effect on the dispersion of GO in cement suspension. Among them, P-HRWR exhibited the best promoting effect, followed by N-HRWR and AEA. After the GO suspension with the addition of AEA precipitated, no GO remained in the upper solution, while the upper GO suspension with P-HRWR still contained a large amount of GO.

#### 3.3.2. Mechanisms of GO Agglomeration in Cement Paste

As indicated by the investigations on the dispersion and agglomeration of GO in aqueous solutions, SCPS and cement pastes, the comparisons of multiple DAs indicated that P-HRWR had the most significant effect of avoiding the dispersion and re-agglomeration of GO in cement pastes.

**Figure 11:**
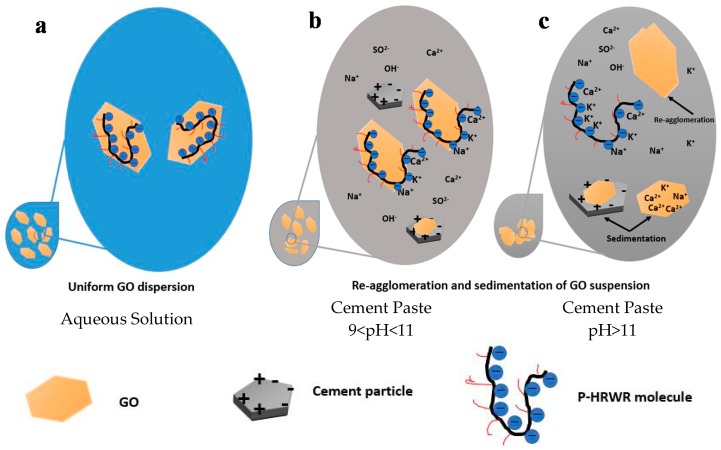
Schematic representation of the mechanisms of destabilization of the GO dispersion in the (**a**) aqueous solution; (**b**) cement paste 9<pH<11; and (**c**) cement paste pH>11 for the P-HRWR.

The influence mechanism of P-HRWR on the agglomeration and sedimentation of GO in solutions with different pH and ion concentrations were examined, as shown in Figure 11. The P-HRWR could be adsorbed onto GO in aqueous solutions and as a result GO was stable to exist in aqueous solutions by virtue of electrostatic interaction and steric hindrance [27,28]. N-HRWR realized the stable existence of GO via electrostatic interaction and AEA realized that through decreasing the surface tension of solutions. 

With the addition and hydration of cement, the pH of the cement pastes continuously increased (under 11). The ceaseless increase of ions from the cement was due to continuous dissolution of cement particles and undissolved particles containing surface charges could adsorb the GO in solutions, resulting in sedimentation. When the pH of the solution reached above 11, the presence of cations in the cement pastes could alter the electrostatic layers surrounding the GOs in the P-HRWR assisted suspensions and allowed for the GOs to bind with cations [22,58] and to come into contact or within the distance in which the Van der Waals forces are dominant. Strong interactions between GO and cement particles lead to the sedimentation and re-agglomerates of GO. For the AEA assisted dispersion, the ions present in the cement pastes were thought to increase the surface tension of the solution [52] and negated the effect of the AEA.

## 4. Conclusions

Based on the systematically designed experiments, the dispersion, re-agglomeration and related mechanisms of GO in aqueous solutions, SCPS and suspension of cement pastes were investigated. The following conclusions concerning GO stability and agglomeration were made:

(1) The dispersion of GO in aqueous solutions was positively influenced by P-HRWA, N-HRWA, and AEA, respectively; among the DAs, the AEA provided the most improvement of dispersion in aqueous solutions.
(2) Compared with the dispersion of GO in SCPS and cement pastes, homogeneous dispersion of GO in aqueous solution did not guarantee uniform dispersion in cement pastes. Although AEA allowed GO to reach the optimum state of dispersion in aqueous solutions, lowering the surface tension of the aqueous solution was AEA’s main advantage, which did not exist in the highly alkaline and high ionic strength solution.
(3) The dual effects of electronic repulsion and steric hindrance of P-HRWR resulted in better GO dispersion in the high alkalinity and high ionic strength of cement paste.
(4) With the increase of ions and pH, the P-HRWR separated from the GO. Thus, GO were absorbed on the surface of the cement particles, and GO sedimentation generated.

## Figures and Tables

**Table 1:** The properties of graphite oxide.

Appearance	Solid Content (mass %)	pH	Viscosity	Absorbance Ratio A230/A600	Carbon (%)	Molar Ratio (O/C)
Brown paste	43 ± 1	≥1.2	≥2000	≥45	47 ± 5	0.6 ± 1

Note: “O”: oxygen atom; “C”: carbon atom.

**Table 2:** The characteristics of DAs.

Type	Aspect	Solid Content, %	pH	Recommended Content
RMC-3	Dark-brown liquid	49.98	5–6	0.5%–1.5%
CP-WRM50	Slight-yellow liquid	50.79	4–5	
KS-20	Yellow brown solid	95	6–7 ^a^	0.75%–1.5%
K12	White solid	96	7–8 ^a^	0.03%–0.1%

Note: ^a^ solution with the mass concentration of 5%.

**Table 3:** Chemical compositions (% by mass) and physical properties of cement.

Composition	CaO	SiO_2_	Al_2_O_3_	Fe_2_O_3_	MgO	SO_3_	f-CaO	Na_2_O	LOI
**Content (%)**	64.65	21.88	4.49	3.45	2.36	2.44	0.28	0.51	1.31
**Fineness (0.08/%)**	**Specific Surface Area (m^2^/kg)**			**Density** **(g/cm^3^)**		**Setting Times (min)**		**Stability**	
0.8	344			3.15		Final	Initial	Qualified	
						130	205		

**Table 4:** DAs to GO mass ratios and corresponding DA to solution mass ratios.

Sample	GO (g)	DAs (g)	Water (g)
pGO	0.03	0	999.97
GO-N-1	0.03	N-HRWR, 0.03	999.97
GO-N-3	0.03	N-HRWR, 0.09	999.97
GO-N-9	0.03	N-HRWR, 0.27	999.97
GO-A-1	0.03	AEA, 0.03	999.97
GO-A-3	0.03	AEA, 0.09	999.97
GO-A-9	0.03	AEA, 0.27	999.97
GO-P-1	0.03	P-HRWR, 0.03	999.97
GO-P-3	0.03	P-HRWR, 0.09	999.97
GO-P-9	0.03	P-HRWR, 0.27	999.97

**Table 5:** Surface parameters and particle size characteristics of GO in aqueous solution.

Sample	Sample Style (Diameter)	Solvent	Particle Weight Concentration	Density of Particle (g/mL)	Specific Surface Area (m^2^/g)
pGO	GO (350 nm)	Water	0.00003	2.2	2112.45
pGO-JZ30min	GO (350 nm)	Water	0.00003	2.2	1824.47
GO-N-1-JZ30min	GO (350 nm)	Water	0.00003	2.2	1954.47
GO-N-3-JZ30min	GO (350 nm)	Water	0.00003	2.2	1978.14
GO-N-9-JZ30min	GO (350 nm)	Water	0.00003	2.2	2001.17
GO-A-1-JZ30min	GO (350 nm)	Water	0.00003	2.2	2025.31
GO-A-3-JZ30min	GO (350 nm)	Water	0.00003	2.2	2077.97
GO-A-9-JZ30min	GO (350 nm)	Water	0.00003	2.2	2021.15
GO-P-1-JZ30min	GO (350 nm)	Water	0.00003	2.2	2000.47
GO-P-3-JZ30min	GO (350 nm)	Water	0.00003	2.2	2003.14
GO-P-9-JZ30min	GO (350 nm)	Water	0.00003	2.2	2217.63

Note: “JZ30min”: resting solution for 30 min before testing.

**Table 6:** Surface parameters and particle size characteristics of GO in simulated concrete pore solution.

Sample	Sample Style (Diameter)	Solvent	Particle Weight Concentration	Density of Particle (g/mL)	Specific Surface Area (m^2^/g)
pGO-JZ30min	GO (350 nm)	Water	0.00003	2.2	54.87
GO-N-3-JZ30min	GO (350 nm)	Water-N	0.00003	2.2	215.47
GO-A-9-JZ30min	GO (350 nm)	Water-A	0.00003	2.2	60.47
GO-P-1-JZ30min	GO (350 nm)	Water-P	0.00003	2.2	1205.35

**Table 7:** Surface parameters and particle size characteristics of GO in suspension of cement pastes.

Sample	Sample Style (Diameter)	Solvent	Particle Weight Concentration	Density of Particle (g/mL)	Specific Surface Area (m^2^/g)
GO-N-3-C-JZ30min	GO (350 nm)	Water-N-C	0.00003	2.2	515.47
GO-A-9-C-JZ30min	GO (350 nm)	Water-A-C	0.00003	2.2	87.95
GO-P-1-C-JZ30min	GO (350 nm)	Water-P-C	0.00003	2.2	1745.12
